# Genetic Polymorphism in the *Amaranthaceae* Species in the Context of Stress Tolerance

**DOI:** 10.3390/plants12193470

**Published:** 2023-10-03

**Authors:** Nina V. Terletskaya, Oxana N. Khapilina, Ainur S. Turzhanova, Malika Erbay, Saule Magzumova, Aigerim Mamirova

**Affiliations:** 1 Faculty of Biology and Biotechnology, Al-Farabi Kazakh National University, Al-Farabi 71, Almaty 050040, Kazakhstan; a.mamirova.95@gmail.com; 2 Institute of Genetic and Physiology, Al-Farabi 93, Almaty 050040, Kazakhstan; malika.isa99@mail.ru; 3 National Center for Biotechnology, Qorghalzhyn 13, Astana 010000, Kazakhstan; turzhanova-ainur@mail.ru (A.S.T.); magzumovas@list.ru (S.M.)

**Keywords:** *Amaranthaceae* species, polymorphism, abiotic stress, superoxide dismutase, auxin response factors, inter-primer binding site amplification profiling

## Abstract

The adaptive potential and biochemical properties of the *Amaranthaceae* species make them promising for introduction into agriculture and markets, particularly in arid conditions. Molecular genetic polymorphism analysis is the most powerful tool for studying plant resources; therefore, the current study aimed to investigate the polymorphisms of allelic variations in the *ARF* and *SOD* gene families, as well as the genetic diversity of six *Amaranthaceae* species, using retrotransposon-based fingerprinting with the multi-locus EPIC-PCR profiling approach. Additionally, the iPBS PCR amplification was employed for genome profiling, revealing variations in genetic diversity among the studied *Amaranthaceae* samples. The observed genetic diversity in *Amaranthaceae* species contributes to their enhanced tolerance to adverse environmental conditions. The knowledge about the genetic diversity of genes crucial in plant development and stress resistance can be useful for the genetic improvement of cultivated *Amaranthaceae* species.

## 1. Introduction

Amaranths, belonging to the *Amaranthaceae* family, are relatively recent additions to contemporary agriculture, originating from the New World. Many members of the family are classified as pseudo-cereals (*Caryophyllales: Amaranthaceae*). They have gained widespread attention in recent years due to their highly nutritious grain and green mass, particularly their valuable amino acid composition (abundant in lysine, arginine, and histidine) [1,2,3]. Amaranth also contains medical bioactive substances such as squalene and antioxidants, which contribute to disease prevention [4,5,6,7]. The substantial iron (Fe) content in amaranth seeds makes them potentially effective in combating Fe-deficiency anaemia [8]. Moreover, amaranth seed protein is gluten-free, making it an excellent protein source for individuals with celiac disease [9,10,11]. Pharmacological studies have revealed various properties of amaranth, including hepatoprotective, radioprotective, anti-inflammatory, antipyretic, antihepatotoxic, antidiabetic, antihyperlipidemic, spermatogenic, antiproliferative, and antifungal effects [4,5,6,7,8,9,10,11,12].

*Amaranthaceae* plants exhibit remarkable resilience to adverse environmental conditions such as nutrient-poor soil, water scarcity, and severe defoliation [13]. Unlike traditional crops, amaranths not only withstand harsh conditions such as drought or soil salinity but also show an increase in proteins, vitamins, phenolic acids, flavonoids, and natural antioxidant content [7,14,15,16]. Therefore, amaranths can be considered a promising choice for farmers, particularly in marginal soils.

Understanding plant responses to environmental stress necessitates comprehensive investigations into physiological, cellular, and transcriptome alterations that ensure plant survival [17]; however, considerable knowledge gaps remain in these regulatory processes. Plant stress tolerance research relies greatly on genetic polymorphism, which underlies the natural diversity of plant—stressor interactions. Analysing the molecular genetic polymorphisms of genes involved in phytohormone activity, isoenzymes, and metabolic pathways provides insights into how plants respond to stress conditions [17,18]. Genetic polymorphism studies offer valuable information from both an evolutionary and genetic-diversity standpoint. According to recent molecular marker-based studies, modern amaranth species are closely related and derived from *A. hybridus* through multiple independent domestication events [19,20,21,22].

Species *A. caudatus*, *A. deflexus*, *A. hypochondriacus*, *A. retroflexus*, *A. spinosis* (epinard), and *Ch. quinoa* (cultivar “Vahdat”) selected for this study are undoubtedly related, originating in Central and South America but with varying degrees of domestication: from malicious weeds like *A. deflexus* [23] and *A. spinosis* (epinard) [24] to forage, vegetable, medicinal, and ornamental species like *A. retroflexus* [25], *A. caudatus* [26], *A. hypochondriacus* [27,28], and *Ch. quinoa* [15,16]. The resistance to abiotic forces may have altered as a result of introduction and domestication.

Polymerase chain reaction (PCR) is commonly employed to assess the genetic diversity of individual amaranth species [29]. Identifying and examining polymorphisms sheds light on the evolutionary paths that have shaped the current state of genes in related species [30].

The literature data show that the phytohormone auxin plays a crucial role in regulating growth and development processes in plants subjected to environmental stressors. This regulation occurs via gene expression control, mediated by a family of diverse DNA-binding ARFs (auxin response factors) [31,32,33,34,35]. EPIC-PCR (exon-primed intron-crossing PCR), a highly accurate and sensitive genotyping method, allows for the concurrent analysis of many samples. Plant growth and development can be influenced by genetic variations in *ARF6*, a key factor in regulating the auxin response. The genetic polymorphism of diverse plant species can be explored using EPIC-PCR primers targeting the *ARF6* gene family [36]; however, understanding of the *ARF* function in plants other than model organisms, such as *Arabidopsis*, is sparse. Comparing genetic polymorphism in auxin response genes across different amaranth species might be useful for studying their genetic traits and evolution [37].

Plants subjected to abiotic stress simultaneously experience oxidative stress. Various biological activities, molecular functions, and cellular components rely heavily on redox reactions. Defence enzymes are actively involved in reducing ROS (reactive oxygen species). Consequently, agricultural crop stress tolerance is largely determined via ROS concentration and defence enzyme activity, particularly SOD (superoxide dismutase). Different SOD isoforms serve as the primary defence line in plants against ROS-induced damage [38,39,40]. In recent years, there has been a growing focus on studying the genetic polymorphism of *SOD* genes in *Amaranthaceae* species [41]. Universal EPIC primers are employed for genetic polymorphism analysis, enabling simultaneous amplification of multiple loci. This approach will provide more comprehensive insights into the genetic diversity within populations of various amaranth species [42].

Molecular genetic polymorphism encompasses genetic variation not only in genome coding regions but non-coding regions as well, which contain a large number of mobile elements in eukaryotes. An examination of repetitive elements in the amaranth genome indicated the presence of Copia- and Gypsy-like retrotransposons [22,43]. These retrotransposons serve as fundamental components in managing epigenetic changes during short-term stressors and may operate as a driving force in species evolution [44,45,46,47,48]. It has been proposed that certain retrotransposons are mainly deployed into genes that are particularly sensitive to environmental changes as a result of evolutionary adaptations [49]. One method for studying genetic polymorphism at the retrotransposon level is to use iPBS (inter-primer binding site) markers, which have proven to be informative and effective in exploring the genetic diversity of *Amaranthaceae* [50,51].

This study aimed to investigate the genetic polymorphism of the *SOD* and *ARF* gene families in the *Amaranthaceae* species, such as *A. caudatus*, *A. deflexus*, *A. hypochondriacus*, *A. retroflexus*, *A. spinosis (epinard)*, and *Ch. Quinoa*, as well as conduct a retrotransposon genetic analysis utilising genomic profiling approaches to elaborate knowledge on the role of the above genetic factors in conferring tolerance to abiotic stressors in *Amaranthaceae* species.

## 2. Results

### 2.1. Genetic Diversity of Auxin Response Factor 6 Gene Family in Amaranthaceae Species

The investigation of genetic diversity within the *ARF6* gene group revealed different levels of allele (*Na*) richness among the plant species analysed. *A. deflexus* had the highest Na value (1.255) among the species, indicating a greater number of distinct alleles in its population. The effective number of alleles (*Ne*) was generally higher than Na, indicating that some alleles were more dominant in certain species. Shannon’s Diversity Index (I) ranged from 0.103 (*A. spinosis*) to 0.266 (*A. deflexus*), the latter having the highest allele frequency diversity.

In the context of *SOD* genes, Na varied across species. The highest Na value of 1.019 was found in *A. deflexus*, illustrating its genetic diversity. The effective allele number (*Ne*) followed the same trends as *Na*, demonstrating that allele frequencies vary. Shannon’s diversity index (*I*) varied from 0.048 for *A. hypochondriacus* to 0.257 for *Ch. quinoa*.

The iPBS profile research indicated a wide range of genetic characteristics among species. The most diverse group was *A. spinosis* (epinard), which had the greatest mean allele count (*Na* = 1.141). The effective allele count (*Ne*) revealed a range of values, reflecting a variety of allele frequencies. Shannon’s Diversity Index (*I*) varied from 0.056 for *Ch. quinoa* to 0.282 for *A. spinosis*, showing varying degrees of genetic diversity. Thus, accessions of *A. deflexus* had the largest effective number of alleles (*Ne*) and percentage of polymorphic loci (%P), indicating potentially substantial genetic diversity (Table 1).

In addition, the *A. spinosis* (epinard) samples exhibit the lowest levels of heterozygosity (*He*) and unexpected heterozygosity (*uHe*) among the other species, indicating a potentially low genetic variability of *A. spinosis*. To assess the overall genetic diversity among all studied Amaranthaceae species, general genetic parameters such as Mean and SE were employed. The percentage of polymorphic loci (*NPB*) for auxin response genes was found to be 39.7%, indicating a relatively high level of genetic diversity across all examined species.

The AMOVA (molecular variability analysis) of the *ARF6* genes provides insights into the structure of the examined amaranth species by dissecting the dispersion of this characteristic into various hierarchical components. Table 2 displays the findings of the AMOVA analysis, indicating that 25% of the genetic variability can be attributed to genetic differentiation between the *Amaranthaceae* species studied.

The remaining 75% of the molecular variance can be attributed to the genetic diversity within the species. The *PhiPT* value, a measure of genetic differentiation among species, was estimated at 0.253, indicating moderate differences between the species investigated.

**Figure 1:**
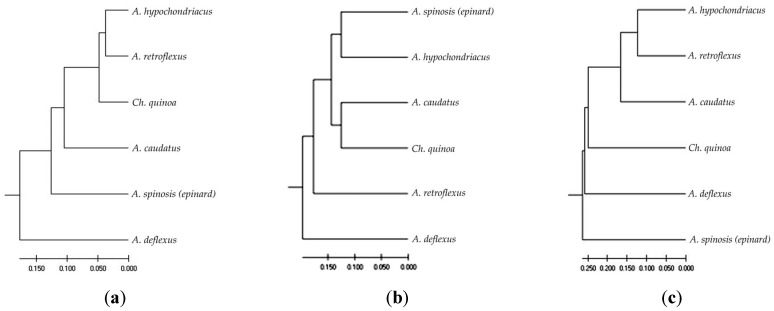
Genetic association of six genomes of *Аmaranthaceae* depending on the similarity–difference in the studied genes and DNA regions: (**a**) *ARF6* genes; (**b**) *SOD* genes; (**c**) iPBS profiling.

Figure 1a displays a dendrogram illustrating the distribution of species based on the evolutionary aspect of similarity and differences in the studied *ARF6* genes and DNA regions. *A. hypochondriacus*, *A. spinosis (epinard)*, and *Ch. quinoa* are the most genetically similar species in terms of auxin response genes, while *A. deflexus* exhibits the highest genetic diversity and differs significantly from the above species.

To analyse the significance of the detected polymorphism and to identify the influence of selection on the diversity of gene nucleotide sequences and the number of dispersed repetitions, Tajima’s statistical test for neutrality was used. The data provided in Table 3 reveal a low level of genetic variability among the investigated *Amaranthaceae* species.

Tajima’s D statistical test allowed us to examine deviations from the neutral evolutionary model of *Amaranthaceae* and demonstrated the presence of a potential effect of selection on genetic variability in the species tested. The percentage of polymorphism (ps) was reported as 0.96, suggesting a high degree of homozygosity in the iPBS loci and antioxidant system genes studied within the *Amaranthaceae* species. Additionally, the values of the parameters Θ and π were low, and the value of D was negative, indicating the presence of positive selection phenomena.

### 2.2. Genetic Diversity of Superoxide Dismutase Gene Family for Amaranthaceae Species

The analysis of variance results (Table 1) indicated that the predominant source of variation (61%) stemmed from disparities in *SOD* genes between *Amaranthaceae* species. On the other hand, differences within the species accounted for 39% of the observed variation.

The amplification of *SOD* gene families was performed on six *Amaranthaceae* species, and the results were analysed using a fingerprinting technique (Table 2). The average values of heterozygosity (*He*) and observed heterozygosity (*Ho*) for all the *Amaranthaceae* species ranged from 0.101 to 0.149, respectively. These findings suggest that the observed heterozygosity exceeds the expected values, potentially indicating the occurrence of intraspecific crossing.

Table 2 also presents the values of Shannon’s coefficient (*I*) and the coefficient of unexpected heterozygosity (*uHe*), which evaluate the diversity of genotypes within species. Notably, the average values of *uHe* were 0.038 in *A. hypochondriacus* and 0.198 in *Ch. quinoa*, demonstrating varying levels of genetic diversity among the investigated species. Consequently, the heterozygosity (*He*) and unexpected heterozygosity (*uHe*) values for *A. hypochondriacus* samples were significantly lower compared to the other species. These results suggest that *A. hypochondriacus* may possess lesser genetic diversity than the other species.

The cluster analysis results (Figure 1b) indicate that the allelic variations in the *SOD* gene were most similar between *A. hypochondriacus* and *A. spinosis (epinard)*, as well as between *A. caudatus* and *Ch. quinoa*; conversely, *A. deflexus* exhibited the greatest genetic diversity, displaying significant differences from the other species.

The neutrality test conducted on the *Amaranthaceae* species, based on the analysis of *SOD* gene polymorphism, yielded statistically significant values (Table 3). The *ps* value, representing the proportion of polymorphic sites, was determined to be 0.962963, indicating a high level of polymorphism within the studied species. The *Θ* value, representing the average genetic distance between randomly selected sequences, was calculated as 0.243071. Additionally, the π value, representing the average number of differences per site, was found to be 0.315411.

Tajima’s D coefficient, which reflects the balance between neutral mutational drift and natural selection, was calculated as 1.111407. This value suggests the presence of balancing selection as a contributing factor.

### 2.3. Inter-Primer Binding Site (iPBS) Genome Fingerprinting Analysis for Amaranthaceae Species

The AMOVA results (Table 1) indicate a substantial level of genetic variability among the investigated amaranth species at the iPBS level. This variability can be attributed to disparities in genetic composition or population isolation. The genetic variance (*PhiPT*) between species was determined to be 49%, indicating that approximately half of the genetic variability was attributable to the differences between species; however, the within-species genetic variability level was also significant at 51%, suggesting the influence of mutations, genetic drift, or selection within the studied species.

The analysis of genetic diversity indicators (Table 2) suggested a relatively high level of genetic diversity among the six amaranth species at the iPBS level. Across all species, the average effective number of alleles (*Ne*) and expected heterozygosity (*He*) ranged from 1.068 to 1.321 and 0.119 to 0.128, respectively. These values indicate a considerable number of alleles and a high degree of genetic variability within the studied species.

Meanwhile, *A. spinosis (epinard)* exhibited the highest number of alleles (1.141) and expected heterozygosity (1.321); conversely, *Ch. quinoa* displayed the smallest number of alleles (0.328) and expected heterozygosity (1.068). The diversity index, observed heterozygosity, and percentage of polymorphism also followed a similar pattern, with *A. spinosis (epinard)* demonstrating the highest values and *Ch. quinoa* displaying the lowest.

Amaranth species with higher observed heterozygosity, such as *A. deflexus* and *A. spinosis (epinard)*, also exhibited elevated levels of expected heterozygosity, indicating their greater genetic diversity. Furthermore, *A. hypochondriacus* and *A. caudatus* showed similar levels of genetic diversity, suggesting potential shared evolutionary pressures or demographic events. In contrast, other species displayed more variation in their levels of genetic diversity. Overall, accessions of *A. spinosis (epinard)* showcased the highest genetic diversity, while accessions of *Ch. quinoa* exhibited the lowest diversity.

The cluster analysis results based on the retrotransposon analysis indicated that *A. retroflexus*, *A. hypochondriacus*, and *A. caudatus* (Figure 1c) exhibit the highest genetic similarity. In contrast, *A. spinosis (epinard)* showed the greatest genetic diversity and differed significantly from the other species.

The neutrality test conducted on the *Amaranthaceae* species revealed that they are not in a state of Hardy–Weinberg equilibrium. This deviation suggests the presence of natural selection and balancing selection. Balancing selection refers to a mechanism that maintains, increases, or regulates genetic variability without the emergence of new forms or morphophysiological adaptations. In the context of balancing selection, the absence of rare alleles is observed. The evenly distributed *D* value further confirms the balance between the frequencies of different alleles, as presented in Table 3.

## 3. Discussion

The speciation of *Amaranthaceae* representatives is a relatively recent evolutionary event [52], offering a valuable opportunity to study the early stages of genome differentiation.

*Amaranthaceae* species exhibit variations in their level of domestication. While species within this family possess valuable macro- and microelements, as well as biologically active substances with medicinal properties in their grains and green biomass, certain species such as *A. spinosis (epinard)*, *A. retroflexus*, and *A. deflexus* are regarded as invasive weeds in numerous regions.

Species within the *Amaranthaceae* family exhibit variations in their chromosome numbers. The majority of recorded species have diploid chromosome numbers, such as 2n = 32 for species like *A. hypochondriacus*, *A. cruentus*, *A. caudatus*, and *A. retroflexus*. Other species, such as *A. spinosis* and *A. deflexus*, have a chromosome number of 2n = 34, while *Ch. quinoa* has a chromosome number of 2n = 36 [22,53].

These observations suggest the occurrence of a whole genome duplication (WGD) event in the *Amaranthaceae* family, which likely took place between 36.7 and 67.9 million years ago. This WGD event may have been accompanied by subsequent stages of chromosome fusion or loss [22]. Overall, amaranths are generally classified as paleo-allotetraploid.

Nowadays, geneticists have made significant progress by utilizing various research approaches and resources in the field of amaranth research. These include the use of genetic markers and genetic linkage maps [22,54,55]. Modern PCR methods, coupled with genome profiling using multicopy and the genomic abundance of transposable elements and endogenous viruses, have contributed to expanding the theoretical knowledge base on phylogenetic relationships and accurately assessing the genetic diversity of particular species [48,56,57,58].

One such approach is genome profiling based on intermittent repeats, which relies on the distribution of transposable elements, particularly long terminal repeat (LTR) retrotransposons, throughout the genome. PCR using a single primer targeting conserved sequences in LTR retrotransposons is an exemplary technique used for interspersed repeat-based genome profiling. Additionally, the iPBS amplification has proven to be a powerful genome fingerprinting technique that does not require retrotransposon sequence information. Furthermore, the palindromic sequence-targeted (PST) PCR enables genome crawling and profiling, facilitating the initial characterization of intra- and interspecific genetic variability, as well as tracking specific lineages and genotypes [59].

Polymorphism, characterized by the presence of multiple variants (alleles) of individual genes within a species, can arise from various factors including mutations, random gene drift, and natural selection [60]. Natural selection has played a crucial role in shaping adaptive traits in plant species [61,62]. Intraspecific genetic variability is essential for species’ survival and reproductive success, enabling them to expand their range and withstand the pressures of natural selection in challenging environmental conditions at the population level [63]. The exploration and identification of polymorphism provide insights into the evolutionary paths that have led to the current genetic composition of related species; therefore, it is vital to consider polymorphism within the context of natural and artificial selection phenomena [31].

To elucidate the nature of polymorphism and evaluate its significance, geneticists have developed various criteria that help assess the presence of selection pressure and identify the type of natural selection occurring. In particular, Tajima’s test is the commonly used statistical test for neutrality, which examines the distribution of allele frequencies of the genes. This test enables a comparison of the average number of nucleotide substitutions and polymorphic sites within a sample [64].

By employing EPIC-PCR, the dominant selection type was determined. The data obtained from the *ARF6* genes, for instance, allowed us to assume the occurrence of positive selection, wherein there was a drive to propagate and fix emerging “beneficial” polymorphisms. This suggests that the species may have undergone expansion at that particular time. In such species, it is common to observe a relatively high frequency of rare “beneficial” alleles, which could contribute to stress tolerance in the studied amaranth species [65].

When the *D*-criterion of Tajima’s test and other parameters, such as those obtained from *SOD* genes and iPBS profiling, exhibit a positive value (*D* > 0), it suggests either a sudden reduction in population size or the influence of disruptive (balancing) selection. This may indicate the presence of excessive heterozygosity in the studied species due to gene flow. In such cases, genetic variability is preserved, increased, or regulated without the emergence of new morphophysiological adaptations or life forms, and rare alleles are typically absent. Balancing selection promotes the expansion of adaptive capabilities within species without the emergence of new forms and contributes to the maintenance of polymorphism [66]. One mechanism underlying balancing selection is the selective advantage of heterozygotes possessing two different alleles over homozygotes carrying only one allele [67].

According to the obtained results, weed species such as *A. spinosis (epinard)*, *A. retroflexus*, and *A. deflexus* demonstrated the highest level of genetic polymorphism, which enhances their adaptability and survival in diverse environmental conditions. For instance, in the case of *A. deflexus*, the percentage of polymorphic loci based on *ARF6* genes was 56.36%, and the observed heterozygosity was 0.171. In contrast, *Ch. quinoa* exhibited lower values of 21.82% and 0.068, respectively. Similarly, for *SOD* genes, *A. deflexus* showed high values of these parameters (38.89% and 0.0149, respectively). iPBS profiling revealed that *A. spinosis (epinard)* had a higher percentage of polymorphic loci at 53.13% and observed heterozygosity of 0.188, while *Ch. quinoa* exhibited lower values of 9.38% and 0.039, respectively. These findings align with the relatively low level of outcrossing observed in cultivated amaranths [22,68]. The evolutionary dendrograms constructed using the unweighted pair group method with arithmetic mean (UPGMA) also suggest the presence of these weed species at the evolutionary base, indicating their strong ability to adapt to environmental changes.

Overall, the studied *Amaranthaceae* species exhibit varying yet significant levels of genetic diversity in the studied genes and retrotransposons. This genetic diversity is likely to play a crucial role in their survival and resilience to future stress factors; furthermore, it provides valuable insights for future genetic breeding efforts aimed at developing improved cultivars of amaranth.

## 4. Materials and Methods

### 4.1. Plant Material

The analysis involved seedlings of six *Amaranthaceae* species: *A. caudatus*, *A. deflexus*, *A. hypochondriacus*, *A. retroflexus*, *A. spinosis (epinard)*, and *Ch. quinoa* (Tajik variety “Vahdat”). The seed material was obtained from the collections of the Novosibirsk Institute of Cytology and Genetics, the Siberian Branch of the Russian Academy of Sciences (ICG SB RAS), and the Centre for Genetic Resources of the Tajik Academy of Agricultural Sciences (CGR TAAS). Each species of plants analysed consisted of 25 randomly selected seedlings, with 7–8 shoots per replicate.

Genomic DNA was extracted from fresh plant leaves using a modified CTAB acidic extraction buffer treated with RNAse A [69]. The extraction buffer consisted of 2% CTAB, 2 M NaCl, 10 mM Na_3_EDTA, and 100 mM HEPES at pH 5.3. For DNA isolation, an equivalent amount of fresh tissue from one of the twenty-five randomly selected seedlings, which were grown under identical conditions, was used for each sample.

### 4.2. Exon-Primed Intron-Crossing Profiling for Genetic Polymorphism Analysis of Auxin Response Factor and Superoxide Dismutase Genes of Amaranthaceae Species

PCR primer pairs were designed in this study utilizing the entire nucleotide sequences of the auxin response factor (*ARF6*) and superoxide dismutase (*SOD*) genes. The sequences were retrieved from Genbank NCBI (http://www.ncbi.nlm.nih.gov; accessed on 1 February 2023) and Ensembl Plants (http://plants.ensembl.org; accessed on 1 February 2023). The primer characteristics for EPIC-PCR amplification of the *ARF6* family genes are provided in Table 4.

Primer pairs 5175–5176 and 5176–5179 exhibit high information content (PPL, %) and diversity (PIC), enabling their utilization in the analysis of genetic polymorphism within the *ARF* family genes. These primers generate amplicons of varying sizes, facilitating the assessment of genetic polymorphism across both large and small gene fragments.

For evaluating the polymorphism of *SOD* gene introns, the primers listed in Table 5 were employed.

The utilization of the polymorphism informativeness coefficient (PIC) in analysing locus polymorphism enables the evaluation of genotype diversity within a species. Our findings demonstrate a moderate level of PIC for both primer combinations, suggesting a high degree of genetic diversity in the *Amaranthaceae* species and the significance of these genes in plant adaptation to diverse environmental conditions. The variations observed in amplicon sizes obtained through the primer combinations may indicate the presence of individual differences in *SOD* genes among different amaranth species.

The PCR reaction was performed in a 25 µL reaction mixture containing 50 ng of DNA, 1 × Phire^®^ Hot Start II PCR (Thermo Fisher Scientific Inc., Waltham, MA, USA) buffer with 1.5 mM MgCl_2_, 1 µL of 10 mM primer, 0.2 µL of a 10 mM dNTP mixture, and 0.2 µL of 1 U Phire Hot Start Polymerase. The amplification protocol consisted of an initial denaturation step at 98 °C for 1 min, followed by 30 cycles of denaturation at 98 °C for 5 s, annealing at 60 °C for 30 s, extension at 72 °C for 1 min, and a final extension step at 72 °C for 3 min. The amplification was performed using a T100 Thermal Cycler (Bio-Rad Laboratories Inc., Hercules, CA, USA).

The amplified products were subjected to electrophoresis on a 1.5% agarose gel, and the bands were visualized using ethidium bromide staining (Figures S1 and S2). The length of DNA fragments was determined by comparing them to a molecular weight marker (Gene-Rules DNA Ladder #SM0333, Thermo Fisher Scientific Inc., Waltham, MA, USA) using the Quantity One program in the PharosFX Plus gel documentation system (Bio-Rad Laboratories Inc., Hercules, CA, USA). The level of polymorphism was assessed by calculating the percentage of polymorphic amplicons out of the total number of amplicons obtained for each primer.

### 4.3. Inter-Primer Binding Site (iPBS) Genome Fingerprinting

The analysis employed universal Primer Binding Sites (PBS) primers, and their characteristics are provided in Table 6. These primers were designed to target the PBS, which are specific sections of retrotransposons.

The PCR reaction was conducted in a 25 µL reaction mixture, consisting of 50 ng of DNA, 1 × Phire^®^ Hot Start II PCR buffer with 1.5 mM MgCl_2_, 1 µL of primer (10 mM), 0.2 µL of a mixture of dNTPs (10 mM), and 0.2 µL of 1 U Phire Hot Start Polymerase. The amplification protocol consisted of a preliminary denaturation at 98 °C for 1 min, followed by 30 cycles of denaturation at 98 °C for 5 s, annealing at 53–55 °C for 1 min, extension at 72 °C for 1 min, and a final extension at 72 °C for 1 min. The PCR was performed using an Applied Biosystems VeritiPro 96-Well Thermal Cycler (Thermo Fisher Scientific Inc., Waltham, MA, USA).

The amplified products were subjected to electrophoresis on a 1.2% agarose gel, and visualization was achieved by staining with ethidium bromide (Figure S3). To determine the length of DNA fragments, a molecular weight marker (GeneRules DNA Ladder, Thermo Fisher Scientific Inc., Waltham, MA, USA #SM0333, 100–10,000 bp) was used. The fragment lengths were determined using the Quantity One program in the PharosFX Plus gel documentation system (Bio-Rad Laboratories Inc., Hercules, CA, USA). The level of detected polymorphism was assessed by calculating the percentage of polymorphic amplicons in relation to the total number of amplicons for each primer.

### 4.4. Statistical Data Processing

The gels were assessed using the fingerprint method, and a binary matrix was compiled based on the presence (1) or absence (0) of each fragment. The matrix was then used to perform Tajima’s neutrality test using the Molecular Evolutionary Genetics Analysis (MEGA-X) program [70]. Key indicators of genetic biodiversity, including the number of alleles, Shannon’s information index (I), and genetic differentiation index (PhiPT), were determined using GenAlex 6.5 and dendrogram was constructed using the UPGMA method [64].

## 5. Conclusions

The analysis of *SOD* allelic variants, *ARF6* genes, and the iPBS profiling of genome non-coding regions revealed genetic variability and adaptability in all studied species of the *Amaranthaceae* family, which are currently actively introduced to agriculture in different regions of the globe. Among the studied species, *A. spinosis* (*epinard*) and *A. deflexus* showed the highest genetic diversity, indicating their greater potential for adaptation to environmental changes and suggesting their presence at the evolutionary base. Species with higher genetic diversity offer a wider range of genetic tools that can be used to enhance the stress tolerance of existing cultivars. These findings are valuable for strategy selection to improve existing *Amaranthaceae* cultivars via hybridization with wild types.

## Figures and Tables

**Table 1:** EPIC-PCR and iPBS profiling polymorphism data for 6 *Amaranthaceae* species.

Scheme 6.	*Na*	*Ne*	*I*	*Ho*	*uHe*	*NPB*
***ARF6* genes**						
*A. caudatus*	1.000	1.264	0.242	0.158	0.176	49.09%
*A. deflexus*	1.255	1.276	0.266	0.171	0.190	56.36%
*A. hypochondriacus*	1.018	1.223	0.222	0.141	0.157	49.09%
*A. retroflexus*	0.764	1.144	0.149	0.093	0.103	34.55%
*A. spinosis* (epinard)	0.582	1.080	0.103	0.060	0.067	27.27%
*Ch. quinoa*	0.455	1.110	0.106	0.068	0.076	21.82%
Mean	0.845	1.183	0.181	0.115	0.128	39.70%
SE	0.053	0.016	0.013	0.009	0.010	5.64%
***SOD* genes**						
*A. caudatus*	0.611	1.119	0.103	0.069	0.077	18.52%
*A. deflexus*	1.019	1.257	0.220	0.149	0.165	38.89%
*A. hypochondriacus*	0.370	1.064	0.048	0.034	0.038	7.41%
*A. retroflexus*	0.815	1.123	0.123	0.078	0.086	27.78%
*A. spinosis* (epinard)	0.593	1.166	0.146	0.098	0.109	25.93%
*Ch. quinoa*	0.981	1.324	0.257	0.178	0.198	42.59%
Mean	0.731	1.176	0.149	0.101	0.112	26.85%
SE	0.048	0.018	0.014	0.010	0.011	5.30%
**iPBS profiling**						
*A. caudatus*	0.734	1.235	0.191	0.131	0.146	32.81%
*A. deflexus*	1.078	1.229	0.228	0.144	0.160	51.56%
*A. hypochondriacus*	0.750	1.205	0.177	0.119	0.132	32.81%
*A. retroflexus*	0.781	1.227	0.189	0.128	0.142	34.38%
*A. spinosis* (epinard)	1.141	1.321	0.282	0.188	0.209	53.13%
*Ch. quinoa*	0.328	1.068	0.056	0.039	0.043	9.38%
Mean	0.802	1.214	0.187	0.125	0.139	35.68%
SE	0.048	0.017	0.014	0.009	0.010	6.5%

Note: *Na*—number of alleles; *Ne*—number of effective alleles per locus; *I*—Shannon’s Information Index; *Ho*—observed heterozygosity; *uHE*—unexpected heterozygosity; *NPB*—number (%) of polymorphic loci; *Mean*—mean value; *SE*—standard error.

**Table 2:** Analysis of molecular variance of 6 *Amaranthaceae* species.

Variability	*df*	*SS*	*MS*	*Est. Var.*	*%*	*PhiPT*
Between species	5	68.133	13.627	1.712	25%	0.253
Within species	24	121.600	5.067	1.067	75%	
Total	29	189.733		6.779	100%	
Between species	5	158.967	31.793	5.625	61	0.605
Within species	24	88.000	3.667	3.667	39	
Total	29	246.967		9.292	100	
Between species	5	162.133	32.427	5.362	49%	0.500
Within species	24	134.800	5.617	5.617	51%	
Total	29	296.933		10.979	100%	
*** *p* < 0.001						

Note: *Df*—number of degrees of freedom; *SS*—the sum of squares; *MS*—mean square; *Est. Var*—variance; *PhiPT*—index of genetic differentiation of species. Statistically significant at *** *p* < 0.001.

**Table 3:** Tajima’s Neutrality test analysis.

*m*	*S*	*ps*	*Θ*	*π*	*D*
*ARF6* genes					
30	53	0.963636	0.243241	0.237910	−0.081915
*SOD* genes					
30	52	0.962963	0.243071	0.315411	1.111407
iPBS profiling					
30	62	0.968750	0.244532	0.319971	1.160841

*Note: m*—number of sequences; *S*—the number of segregating sites; *ps*—S/n; *Θ*—ps/a1; π—nucleotide diversity; *D*—Tajima’s test statistic.

**Table 4:** Universal primers used in polymorphism analysis of *ARF* gene family’s allelic variations using multi-locus EPIC-PCR profiling.

ID	Sequence (5′-3′)	Tm, °C *	CG, %	TL	PL	PPL, %	PIC	Amplicon Size, bp
5175-5176	AYTTYCCACARGGYCACAGTGARCA GTCAACAAATACAAGCTGCCAGCCTGATCT	69.3	50.0	171	61	35.68	0.66	250–5000
5176-5179	TCACAYTGGCGNTCAGTNAAGGTT GTCAACAAATACAAGCTGCCAGCCTGATCT	67.6	47.9	149	53	35.57	0.52	400–4000

Note: * Tm—melting temperature, calculated with 1µM concentration and with 1.5 mM Mg^2+^; Ta—optimal annealing temperature; TL—total loci number; PL—polymorphic loci number; PPL—polymorphism percentage; PIC—primer informativeness index.

**Table 5:** Universal primers used in polymorphism analysis of *SOD* gene family’s allelic variations using multi-locus EPIC-PCR profiling.

ID	Sequence (5′-3′)	Tm, °C *	CG, %	TL	PL	PPL, %	PIC	Amplicon Size, bp
5069-5073	CGGAGGCTCTCCAAGGTCGTSTCC AAGCCTCSGCGCGCATCATGCGTA	73.3	66.7	241	72	29.88	0.33	100–4000
5069-5075	CCGGAGGCTCTCCAAGGTCGTSTCC TCCCACAAGTCTAGGCTGATGATTGG	68.9	51.9	292	141	44.18	0.35	150–4000

Note: * Tm—melting temperature, calculated with 1 µM concentration and with 1.5 mM Mg^2+^; Ta—optimal annealing temperature; TL—total loci number; PL—polymorphic loci number; PPL—polymorphism percentage; PIC—primer informativeness index.

**Table 6:** PBS primers used in genetic polymorphism analyses of *Amaranthaceae* species.

ID	Sequence (5′-3′)	Tm, °C *	CG, %	TL	PL	PPL, %	PIC	Amplicon size, bp
2221	ACCTAGCTCACGATGCCA	63.0	50.0	55.4	180	85	49	0.478
2232	AGAGAGGCTCGGATACCA	63.4	55.6	55.4	153	62	40	0.433
2240	AACCTGGCTCAGATGCCA	65.6	55.6	55.0	173	16	10	0.498

Note: * Tm—melting temperature, calculated with 1 µM concentration and with 1.5 mM Mg^2+^; Ta—optimal annealing temperature; TL—total loci number; PL—polymorphic loci number; PPL—polymorphism percentage; PIC—primer informativeness index.

## Data Availability

Not applicable.

## Supplementary Materials

The following supporting information can be downloaded at: https://www.mdpi.com/article/10.3390/plants12193470/s1, Figure S1: Electrophoretic pattern of EPIC-PCR for Amaranthaceae species using different pairs of primers to Auxin Response Factors; Figure S2: Electrophoretic pattern of EPIC-PCR for *Amaranthaceae* species using pairs of primers to conserved regions of genes *SOD*; Figure S3: Electrophoretic pattern of EPIC-PCR and iPBS profiling for *Amaranthaceae* species.
