# Supplementary material for: Genetic Polymorphism in the Amaranthaceae Species in the Context of Stress Tolerance
Source: Plants (Basel). 2023 Oct 3;12(19):3470. doi: 10.3390/plants12193470 (PMC10575142; doi:10.3390/plants12193470)
Supplement: Supplementary file 1 [file plants-12-03470-s001.zip › plants-2551725-supplementary.pdf]

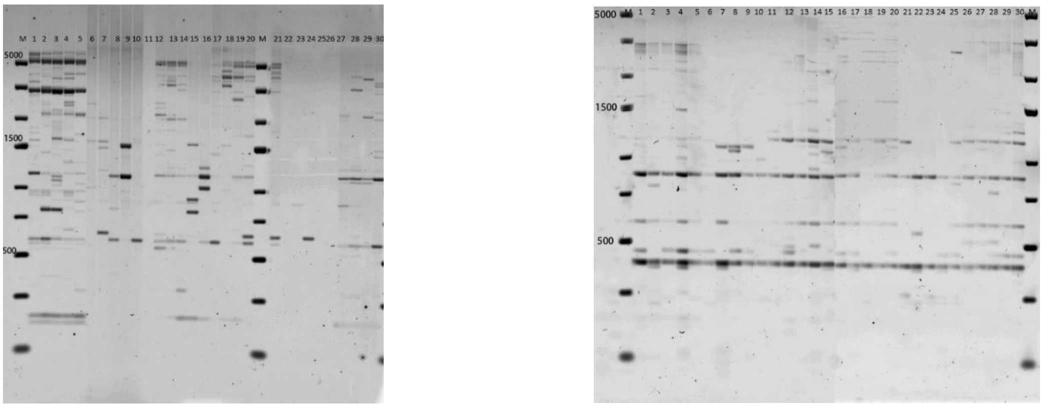

A B

Figure S1. Electrophoretic pattern of EPIC-PCR for *Amaranthaceae* species using different pairs of primers to Auxin Response Factors: (A) праймер 5176-5179: *A. deflexux* 1-5; *Ch. quinoa* 6-1; *A. hypochondriacus* 11-15; *A. caudatus* 16-20; *A. spinosis* (*epinard*) 21-25 *A. retroflexus* 26-30; (B) primer 5175-5179: *A. deflexux* 1-5; *Ch. quinoa* 6-1; *A. hypochondriacus* 11-15; *A. caudatus* 16-20; *A. spinosis* (*epinard*) 21-25 *A. retroflexus* 26-30; M- Thermo Scientific GeneRuler DNA Ladder Mix (100-10,000 bp).

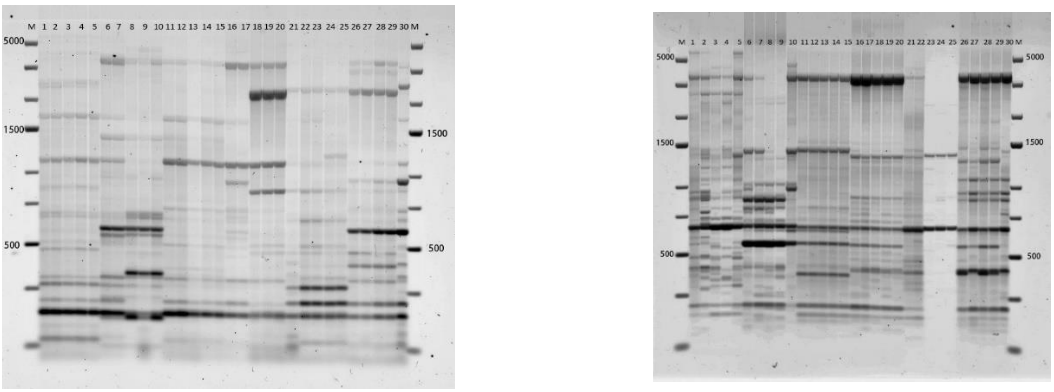

A B

Figure S2. Electrophoretic pattern of EPIC-PCR for *Amaranthaceae* species using pairs of primers to conserved regions of genes SOD: (A) праймер 5176-5179: *A. deflexux* 1-5; *Ch. quinoa* 6-1; *A. hypochondriacus* 11-15; *A. caudatus* 16-20; *A. spinosis* (*epinard*) 21-25 *A. retroflexus* 26-30; (B) primer 5175-5179: *A. deflexux* 1-5; *Ch. quinoa* 6-1; *A. hypochondriacus* 11-15; *A. caudatus* 16-20; *A. spinosis* (*epinard*); 21-25 *A. retroflexus* 26-30; M - Thermo Scientific GeneRuler DNA Ladder Mix (100-10,000 bp).

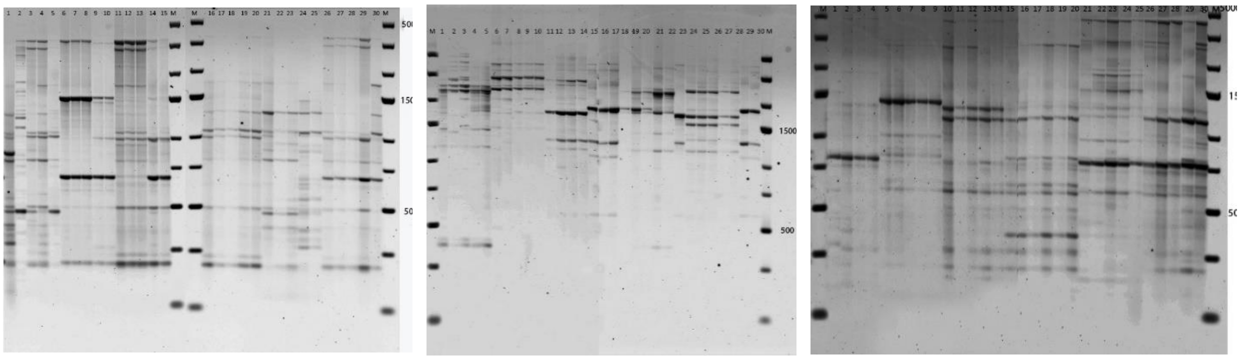

A B C

Figure S3. Electrophoretic pattern of iPBS profiling for *Amaranthaceae* species using single PBS primer: (**A**) primer 2221: *A. deflexux* 1-5; *Ch. quinoa* 6-1 *A. hypochondriacus* 11-15; *A. caudatus* 16-20; *A. spinosis (epinard)*21-25 *A. retroflexus* 26-30 ; (**B**) primer 2232: *A. deflexux* 1-5; *Ch. quinoa* 6-1; *A. hypochondriacus* 11-15; *A. caudatus* 16-20; *A. spinosis (epinard)* 21-25 *A. retroflexus* 26-30; (**C**) primer 2240: *D A. deflexux* 1-5; *Ch. quinoa* 6-1; *A. hypochondriacus* 11-15; *A. caudatus* 16-20; *A. spinosis (epinard)* 21-25 *A. retroflexus* 26-30; M - Thermo Scientific GeneRuler DNA Ladder Mix (100-5000 bp)
